# Immunopathological response of leukocytes against microfilariae and adult worms in white rats infected with *Setaria cervi*

**DOI:** 10.14202/vetworld.2017.562-568

**Published:** 2017-05-30

**Authors:** Sharba Kausar, Wajihullah Khan

**Affiliations:** Department of Zoology, Section of Parasitology, Faculty of Life Sciences, Aligarh Muslim University, Aligarh - 202 002, Uttar Pradesh, India

**Keywords:** differential leukocyte count, liver enzymes, microfilariae, pathology, Setaria cervi, white rats

## Abstract

**Aim:**

Aim of this study was to see the immunopathological changes against the microfilariae (Mf) and adult worms of a bovine filarid, Setaria cervi in the tissues of vital organs of experimentally infected white rats. The effect of diethylcarbamazine (DEC) was also observed on the Mf, as leukocytes especially lymphocytes produce immunoglobulins which opsonize and increase the efficacy of DEC against circulating Mf. Effect of this drug was also assessed on liver enzymes in the microfilaremic rats.

**Materials and Methods:**

Microfilaremia was established by implanting adult worms intraperitoneally and by the infusion of Mf recovered from the uterus of gravid female worms. DEC was administered orally for six consecutive days in the rats having patent infection. Differential leukocyte count was recorded every 3^rd^ day, and liver enzymes were estimated every 10^th^ day in both treated and untreated rats. Pathological changes were observed in HE stained sections of vital organs where Mf or adult worms were trapped.

**Results:**

Destruction and reduction in microfilarial density were noticed in microfilaremic rats treated with DEC. Trapped Mf and embedded worms revealed heavy cellular infiltrations by defensive cells which surrounded and attached with the body surface of the Mf as well as adult worms for their destruction and piece meal clearance. Immune-mediated pathology was observed in the tissue sections of lungs, spleen, and liver. Liver enzymes were elevated during the period of higher parasitemia.

**Conclusions:**

There was a moderate level of immunopathology against the Mf and adult worms by the leukocytes in experimentally infected microfilaremic rats. Mf were in the process of degeneration where they got trapped. Moderate increase in liver enzyme was noticed which was slightly more in untreated group. Although a fraction of Mf gets killed in the peritoneum, majority of them successfully enter the systemic circulation and survive for about 54 days, which is sufficient enough for conducting immunological and chemotherapeutic studies.

## Introduction

Among parasitic diseases, filariasis in man and livestock is a major health problem in tropical countries. The debilitating effects and economic losses caused by these infections severely affect man and animal power resources in developing countries [1-3]. *Setaria cervi* is a cosmopolitan bovine filarid inhabiting the peritoneal cavity of buffaloes (*Bubalus bubalis*). This infection is very common in India showing incidence as high as 70% [4]. The parasites are generally considered to be nonpathogenic in their natural hosts, but transmission of infective larvae through vectors to unnatural hosts, such as goats, sheep or horses, may result in serious and often fatal neuropathological disorders known by the term “cerebrospinal nematodiasis” [5,6]. Larvae of *S. cervi* which normally parasitize in the deer peritoneal and thoracic cavities had been found in the cerebrospinal cavity resulting in lumbar paralysis of the hosts [5-7].

Animal models of filariasis have been used widely for understanding the pathogenesis of the disease, host response, drug screening, and other biochemical aspects [4]. *S. cervi* which has resemblance with human filarids in having similar microfilarial periodicity and a few antigenic components, was chosen for this study. Every host remains in a constant danger of infection caused by pathogens. Host defense against the pathogens is well regulated through inflammatory response marked by leukocyte migration to the site of infection, destruction of the microorganisms and finally, healing and repair of the affected tissue [8]. Most of the helminth parasites including filarids by virtue of being large in size and invasive in nature provide antigenic stimulus to the host to produce antibodies to counter the infection. During their invasion, granulocytes such as eosinophils, basophils, neutrophils along with monocytes play an important role. However, after establishment and chronicity, lymphocytes get activated and start secreting antibodies to counteract the infection [9].

Keeping above findings in view, microfilarial density, differential leukocyte count (DLC), and host reaction by the inflammatory cells were observed in the invaded tissue of experimentally infected white rats before and after the diethylcarbamazine (DEC) treatment. Walling up operation of the invaded worm, destruction and its piece meal clearance was also observed by the defensive cells in the infected tissues of white rats implanted with *S. cervi*.

## Materials and Methods

### Ethical approval

The animals used during the present study were taken from the animal house which maintains all the norms and is registered vide no. 714/02/a/CPCSEA.

### Establishment of microfilaremic rats

Adult *S. cervi* worms were collected from the peritoneal cavity of freshly slaughtered buffaloes (*B. bubalis*) and washed thoroughly in Ringer’s solution at 37°C to remove any debris. 20 laboratory-bred white rats (*Rattus norvegicus*), weighing 125-150 g were used for this study. In a group of 5 rats adult worms were implanted intraperitoneally to see host reaction. Rats were anesthetized and hairs on the lower abdomen were removed with razor. A small incision was made on the left side after cleaning it with alcohol swab and 5 adult worms were introduced into the peritoneal cavity. Cut was stitched with sterilized suture of cat gut and betadine was applied on the wound. Microfilariae (Mf) recovered from the uterus of gravid female worms were infused in the peritoneum of 15 rats with the help of syringe and needle to establish microfilaremia. DEC was given to 10 rats orally at the rate of 100 mg/kg body weight for 6 days after the appearance of Mf in peripheral circulation (i.e., 10^th^ day onward). The effect of DEC was observed on the Mf in the peripheral blood and immunohistopathology in the tissues of vital organs such as liver, lungs, and spleen. After patent microfilaremia, DLCs were recorded every 3^rd^ day from peripheral blood of infected rats to see the changes in the blood picture in treated as well as untreated rats during the course of infection. Peritoneal fluid was also aspirated aseptically, smeared and stained with Leishman’s stain to observe the host response against the Mf in the peritoneum. To study liver enzymes’ activity, blood samples were collected from the tail of rats from 0 to 40^th^ day of infection at 10 days interval. Blood was kept at room temperature for 30 min and then transferred to the refrigerator at 4°C for 2 h so that serum could be squeezed out from the clotted blood. Then, it was transferred to 1.5 ml vials and centrifuged at 1000×*g* at 4 °C for 5 min. Serum was collected and stored at −80°C until used.

### Histopathology

Tissues such as mesentery, lungs, liver, and spleen were collected at necropsy from microfilaremic rats and fixed in Bouin’s solution for 24 h. Fixed tissues were washed, dehydrated and cleared in xylene and finally embedded in paraffin wax. Tissue blocks were trimmed and sectioned at 5 µ. These sections were stretched, dried, dewaxed in xylene. Now sections were hydrated and stained with hematoxylin followed by dehydration up to 70% alcohol and staining in eosin. The stained sections were dehydrated in absolute alcohol, cleared in xylene, mounted in dibutyl phthalate in xylene, dried at room temperature and observed under the Nikon E 600 research microscope to study the pathological changes.

### Biochemical analysis

Liver enzymes such as alkaline phosphatase (ALP), aspartate aminotransferase (AST), and alanine aminotransferase (ALT) were estimated by using Kits from Span, India, according to the manufacturer instructions. AST and ALT were estimated by 2, 4-DNPH method, while ALP was measured by Kind and King’s method in the sera collected from treated and untreated microfilaremic rats with the help of double beam UV-VIS spectrophotometer MODEL LT-2802.

### Statistical analysis

The data were analyzed statistically using one-way analysis of variance and pair wise means were compared using Duncan’s multiple range test (p=0.05). The analysis was performed with the software R Ver 2.14.1.

## Results

**Figure-1:**
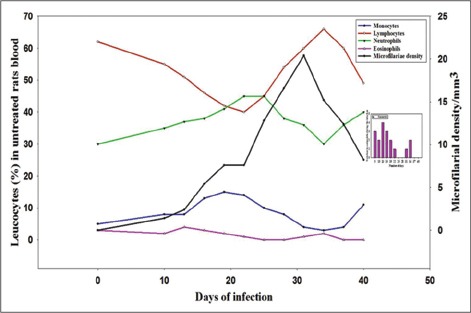
Differential leukocyte count and microfilarial density during the course of infection in untreated rats.

**Figure-2:**
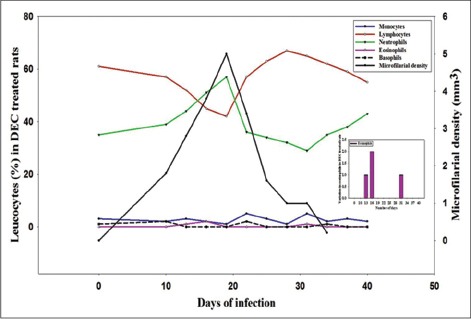
Differential leukocyte count and microfilarial density in diethylcarbamazine treated rats.

Mf appeared in peripheral blood circulation after 8±2 days of initial infection with the intraperitoneal infusion of uterine Mf which continued to persist for 54 days. Maximum microfilarial density recorded was 20/mm^3^ on the 31^st^ day of infection followed by a declining trend which ultimately led to disappearance of Mf on day 55. Results regarding DLCs are shown in graphs (Figures-1 and 2). In infected DEC treated rats, eosinophils and neutrophils showed an elevated picture in DLC with a peak around 16^th^ and 19^th^ day which was little different and recorded on 13^th^ and 25^th^ day in untreated rats, indicating the active involvement of neutrophils for slightly longer duration to counter the infection. As for lymphocyte is concerned, its maximum number was recorded on 28^th^ day post infection in treated rats, while its peak was observed on 34^th^ day in infected control. Early increase of lymphocyte in treated rats was indicator of the fact that these cells were stimulated, multiplied and started secreting antibodies against the circulating Mf. Like eosinophils, monocytes also showed early increase in both treated as well as untreated infected rats which was more prominent in treated group and showed its active involvement in walling up operation and destruction of the parasite.

**Figure-3:**
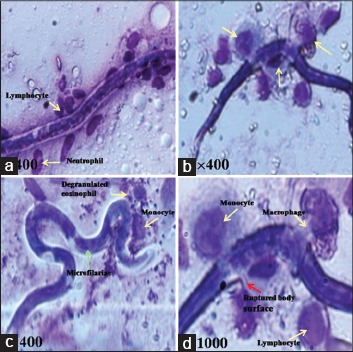
Microfilaria (Mf) of *Setaria cervi* in the process of cell adhesion and destruction by the leukocytes in the peritoneal exudate shown by yellow arrows. (a) Mf surrounded by the leukocytes. (b) Ruptured body surface of Mf along with monocytes, macrophage, and lymphocyte. (c) Mf along with few leukocytes. (d) Magnified version of b showing ruptured surface of Mf shown by red arrow along with attached macrophage, monocytes, lymphocyte, and degranulated eosinophil.

**Figure-4:**
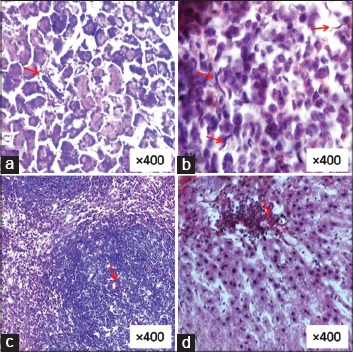
Sections of different vital organs showing trapped microfilariae (Mf). (a) Mesentery with fragment of Mf and few leukocytes in the vicinity. (b) Lung tissue with parts of Mf along with few inflammatory cells. (c) Spleen showing granulomas of leukocyte around the transverse section of Mf. (d) Liver tissue showing intact mf surrounded by inflammatory cells.

The stained smears prepared from the peritoneal exudates showed attachment of monocytes, lymphocytes, and eosinophils around and on the Mf (Figure-3). Mf was distributed more extensively as they were present in blood circulation. After penetration through mesenteries Mf reached in liver, lungs, spleen and other parts of the body through systemic circulation and pathological changes were seen at the sites where they got trapped. In a few cases, these trapped Mf were in the process of destruction due to the inflammatory reactions of defensive cells. Section of mesentery showed fragments of Mf with few defensive cells (Figure-4a). In the lung tissue, Mf which was cut during sectioning showed inflammatory cells in the vicinity (Figure-4b). Aggregation of defensive cells was observed in spleen where lymphocytes were the predominant cells around the transverse section of Mf (Figure-4c). Intact Mf was found in the section of rat’s liver which was surrounded by defensive cells (Figure-4d). Granuloma of inflammatory cells was also present around it.

**Figure-5:**
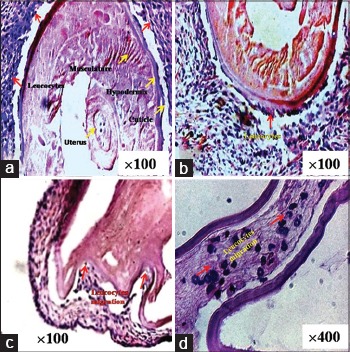
Destruction of trapped *Setaria cervi* worm by the inflammatory cells of white rats. (a) Infiltration and aggregation of leukocytes around the encapsulated worm. (b) Leukocyte adhesion on the cuticle. (c) Adhesion and inpocketing by the leukocytes on the body. (d) Plug formation and migration of the leukocytes within the body.

The infected rats were sacrificed at 5 days interval to recover the worms. 100% live worms were recovered from the peritoneal cavity after 10 days. Except few live worms, most of them were dead and in the process of degeneration on 15^th^ day. Inflammatory patches were visible on the mesenteries, lungs, liver, and spleen due to their encroachments. Dead worms were also found embedded in the peritoneal wall. Hematoxylin and eosin stained tissue sections of mesentery revealed infiltration of inflammatory cells around the embedded adult worms. These sections showed walling up operation where leukocyte infiltration around the worm was clearly visible (Figure-5a). Histological sections revealed aggregation of defensive cells specially monocytes, macrophages, neutrophils, and lymphocytes which started sticking to the cuticle of the worm (Figure-5b). Since parasites were trapped in the tissue permanently, a plug of defensive cells was formed which migrated within the body of the parasite to destroy and clear it off slowly (Figures-5c and d). Worms were encapsulated but not calcified as study was restricted to 40 days. There is a possibility of calcification at a later stage as the parasite was within the capsule formed from the host tissue. Since the parasite was trapped in host tissue for a longer duration, cell infiltration increased. Macrophage, neutrophils, and lymphocytes were the predominant cell types surrounding the worm.

**Figure-6:**
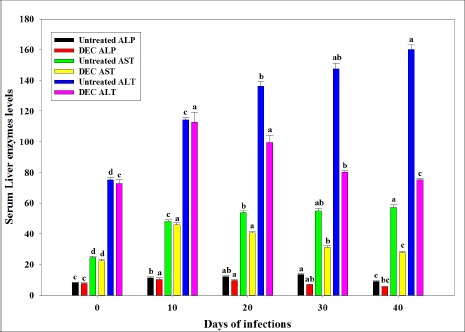
Levels of liver enzymes (alkaline phosphatase [ALP], aspartate aminotransferase [AST] and alanine aminotransferase [ALT]) in the sera of diethylcarbamazine treated and untreated microfilaremic rats (ALP expressed in KA units; AST and ALT in IU/L units).

The concentration of liver enzymes in treated and untreated rats are shown in Figure-6. Liver function tests performed from the sera collected from all the microfilaremic rats showed an increased level of all the enzymes such as AST, ALT, and ALP on 10^th^ day. From 20^th^ day onward concentration of these enzymes get decreased substantially which continues till 40^th^ day of infection in DEC treated rats. However, these enzymes showed significant increase in untreated rats.

## Discussion

*S. cervi* worms gets encapsulated occasionally in the mesenteries and peritoneal walls of buffaloes. In the white rats too when they were implanted in the peritoneum, few worms were found embedded in the mesenteries. Similar penetrations and encapsulations by *S. cervi* were observed in bovines in the patches of inflammatory tissue of the mesenteries, liver, and peritoneum [1]. Almost identical type of host-parasite relationship exists in rat *-S. cervi* model, where the worms were found embedded in the mesenteries and revealed aggregation of inflammatory cells around the worm. Similar reactions from the host were noticed around the Mf, which were infused in the peritoneum. The eosinophils and monocytes were the first to register their increase around the worm as well as Mf and at a later stage they start sticking to the body surface so that they can damage that particular part of the parasite by their secretions and could clear it off in due course of time. When the stay of the parasite was prolonged, neutrophils too became active in the process of destruction along with macrophages and lymphocyte. During the last phase of infection lymphocyte dominated, as they got sensitized at an early stage, multiplied and converted into plasma cells to secrete antibodies which ultimately involved in destruction of the parasite. Eosinophils, lymphocytes, and macrophages were the predominant cell type surrounding the worm and Mf. Similar sequences of destructions of *Setaria cervi, S. digitata*, and *S. tundra* were recorded in buffaloes, reindeer calves and experimentally infected rats and rabbits by earlier workers in Italy, Finland and India [10-13]. Aggregation of inflammatory cells was also somewhat similar around *Litomosoides carinii* which was found embedded in the tissues of experimentally infected white rats, cotton rats, and multimammate rats [14,15].

During this study when hematoxylin-eosine stained sections of mesenteries, lungs, liver, and spleen were examined, few Mf were observed as transverse or oblique sections. Except mesenteries where infiltration by defensive cells was least, there was moderate to intense leukocyte infiltration in the lungs and spleen. Probable reasons for this may be that the Mf find their way to the venules quickly in the mesenteries and reach in portal circulation, while in lungs they stay for a brief period and perforate the endothelial wall to reach in lung alveoli which resulted in lung inflammation. Finally, these Mf if dead were dumped in spleen where plenty of lymphocytes surround and destroy them. The demonstration of Mf in the lungs of infected rats in this study indicated that this filarial infection is a cause of tropical pulmonary eosinophilia (TPE) which was evidenced by nasal discharge, is in agreement with the findings of earlier workers who observed TPE along with emphysematous, degenerative, necrotic and infiltrative changes and exudation in response to *S. cervi* infection in rabbits [16]. The presence of only few trapped Mf in the section of lungs may be due to the reason that most of them might have been destroyed by antibody-dependent, cell-mediated cytotoxicity involving eosinophils. The degenerating Mf also release somatic allergens that bind specific cell-bound immunoglobulin E and thereby trigger the release of vasoactive and inflammatory molecules by lung basophils and mast cells. Almost similar observations were recorded by earlier workers in filarial and other helminthic infections [17-19].

In our study, liver and spleen damage by the Mf were noticed as liver sinusoids were dilated along with increased Kupffer cells. For the long-term chronic helminth infections, the modified Th2 response appears to be an adaptive phenomenon which limits on parasite burden and pathology for persistent infection [20-22]. Destruction of Mf in liver and spleen were observed in the present study which was evidenced by the aggregation of inflammatory cells which restricted and began to destroy them as earlier observed against the Mf of bancroftian filarid where Mf were trapped and destroyed by leukocytes [23,24].

The demonstration of Mf in liver and spleen lesions indicated that filarial infection is a possible cause of hepatomegaly and splenomegaly with eosinophilia. Histopathological examination of spleen of man and monkey showed similar eosinophilic tissue reaction in the form of granulomas around the Mf of *Wuchereria bancrofti* [25,26]. Infiltration of mononuclear cells and polymorphs around the trapped Mf showed moderate host reaction in the rat-*S. cervi* system. Granulomas containing eosinophils with a few lymphocytes and plasma cells around the Mf in the tissue section of spleen were also observed earlier in wild-caught cynomolgus monkeys [27].

Eosinophils, monocytes, macrophages, and neutrophils play a significant role as early defense in the host against the parasites. In microfilaremic rats, we first observed elevation in the count of eosinophils and monocytes, followed by the neutrophils during the early phase. These cells started declining from the peripheral circulation afterwards, indicating their infiltration and migration which can be attributed towards the destruction and elimination of the parasites in the organs where Mf and or adult worms were trapped. In the deeper invaded tissues, these defensive cells might have been associated with piece meal clearance of the parasite as earlier observed in bancroftian filariasis where large numbers of eosinophils were found associated with DEC-induced worm [23]. Eosinophils which are terminally differentiated granular leukocytes reside primarily in vertebrate’s mucosal tissue and function in host defense. These are the first cells involved in the inflammation and histopathology in response to helminth infections. During parasitic infections, the increase in the number of peripheral blood is driven by Th2-derived cytokines, i.e., IL-5, IL-3 [28]. The cationic proteins of the eosinophils damage target cells through membrane interaction as they have the property to disrupt cell membrane. During this study, it was quite evident that eosinophils, monocytes, and lymphocytes eventually targeted the Mf whether it was in the peritoneum or trapped in the viscera. Similar attack by these leukocytes was observed against the Mf of *Onchocerca* and *Brugia* by earlier workers [27,29-30]. Increase in lymphocytes clearly indicated towards the immunological defense which was evidenced by increased numbers of lymphocytes in the peripheral circulation in later phase of infection suggesting the formation of antibodies to neutralize the Mf. In the DEC treated microfilaremic rats, peak concentrations of neutrophils and lymphocytes were little earlier if compared with experimental control rats which might be the effect of opsonization of these defensive cells and their involvement in early destruction and clearance of Mf. Almost similar sequence of events was observed in *Onchocerca* infection [31]. Increase in lymphocytes in treated rats might have been due to the proliferation and transformation of lymphocytes in plasma cells which secrete antibody to eliminate the parasite from infected rats. Similar increase in lymphocyte proliferation was recorded in mice infected with *Nocardia brasiliensis* when treated with DEC [32].

In DEC treated microfilaremic rats, levels of ALP, AST, and ALT enzymes were decreased when compared with untreated rats. Level of liver enzymes was proportionate to the decrease in microfilarial density in treated rats. Since there was a progressive decrease in the microfilarial density in response to DEC treatment, damage of liver cells was minimized and therefore, leakage of enzymes from the liver cells too was less. It is a known fact that DEC interferes with the metabolism of arachidonic acid and its product plays a significant role in liver injury. In addition to that, DEC blocks the production of prostaglandins, resulting in capillary vasoconstriction and infringement in the passage of Mf as observed earlier in carbon tetrachloride-induced liver damage in rats [33]. A noticeable level of increase of ALP, AST and ALT in untreated rats indicated necrotic degeneration of liver cells caused by Mf and possibly the obstruction of bile ducts. Similar necrotic degeneration was observed earlier in man infected with *W. bancrofti* and other helminths [34-36].

## Conclusion

DEC treatment reduced the density as well as the longevity of the Mf in white rats infected with *S. cervi*. During early phase of infection eosinophils, basophils, monocytes, and neutrophils invaded the tissues and enveloped the trapped Mf as well as adult worms for piece meal destruction. During the late phase, there was an increase in lymphocyte which produced secretory antibodies to restrict and destroy the parasite. Mf were found in the process of degeneration in the peritoneal exudates. Immune mediated pathology was also observed around the Mf which were trapped in the tissue sections of lungs, spleen and liver. TPE and nodular lesions were observed in the spleen. Liver enzymes were elevated during the period of higher parasitemia which declined after the treatment of microfilaremic rats with DEC and coincides with reduced microfilarial density. Therefore, it may be concluded that this model invoked moderate immunopathological response against both adult as well as Mf in the tissues where they got trapped, as it is not a natural host. However, since the pathogenicity is not severe, it support the survival of good numbers of Mf in the peripheral blood of *S. cervi*-white rat model for about 2 months, it may prove to be an excellent model for immunopathological and chemotherapeutic studies.

## Authors’ Contributions

SK and WK designed the study. Laboratory work was done by SK. SK has done sampling and analyzed the data. Manuscript was prepared by SK and WK. Both the authors read and approved the final manuscript.
